# Evidence-Based Medicine in the Field of Ophthalmology during the COVID-19 Pandemic

**DOI:** 10.1155/2022/3539134

**Published:** 2022-03-30

**Authors:** Eyal Walter, Brice Vofo, Alan Jotkowitz, Jaime Levy

**Affiliations:** ^1^Department of Ophthalmology, Hadassah Medical Organization and Faculty of Medicine, Hebrew University of Jerusalem, Jerusalem, Israel; ^2^Department of Internal Medicine, Soroka University Medical Center, Ben-Gurion University of the Negev, Beer-Sheva, Israel

## Abstract

**Purpose:**

To describe the evolution of COVID-19 related publications in the field of ophthalmology.

**Methods:**

All articles published in the field of ophthalmology and relevant to COVID-19 were identified by conducting a search on PubMed and Scopus databases using the string ((ophthalmology) OR (eye) OR (ocular)) AND ((corona) OR (COVID-19) OR (pandemic)). Search was conducted on September 30, 2020. Each eligible publication was independently graded by two experienced ophthalmologists based on the level of evidence-based medicine (EBM), with scores ranging from 1 (the highest level of EBM) to 5 (the lowest level). The average level of EBM was also evaluated for each month from February through September. Finally, we analyzed the interval (in days) between submission and acceptance for publication as well as the percentage of manuscripts that required revision before being accepted.

**Results:**

Our search yielded a total of 425 relevant publications. Of these publications, 359 (84.5%), 59 (13.9%), and 7 (1.6%) were rated as level 5, 4, and 3, respectively; none of the publications was rated as level 1 or 2. From February 2020 through September 2020, we found a significant increase in the relative proportion of level 3 and 4 publications compared to level 5 publications (rho = 0.108, *p*=0.024). Moreover, the number of citations per article was significantly correlated with the level of EBM (rho = 2.44, *p* < 0.0005); however, we found no correlation between the number of citations and either the month of publication or the ranking of the journal in which the article was published. The mean interval between submission and acceptance for publication was 20.4 days (SD: 20.2 days), and 48.2% of submitted manuscripts were accepted without revision. From February through September, the interval between submission and acceptance increased significantly (rho = 0.515, *p* < 0.0005); however, we found no significant change in the percentage of publications that were accepted without revision over this same time period.

**Conclusions:**

In the early months of the COVID-19 pandemic, primarily lower-level EBM articles were published, and these publications were accepted relatively quickly. However, this effect was temporary, and over time the EBM levels improved and the interval between submission and acceptance increased, indicating an increase in publication standards.

## 1. Introduction

Evidence-based medicine (EBM) is a well-accepted approach used to address the plethora of information in the field of medicine [1]. In this approach, each new block of information that adds to the overall body of knowledge is categorized using a pyramid—or hierarchy—with studies that have more power and lower bias having more impact [2].

An inherent feature of EBM is that much like a pyramid, higher levels of EBM rely on the lower levels; thus, higher quality EBM is not available in the early stages of an outbreak or pandemic such as the global COVID-19 pandemic [3].

Consistent with reports in other fields of medicine [4–6], we previously found that publications in the field of ophthalmology tended to contain lower levels of EBM during the COVID-19 pandemic compared to pre-COVID-19 publications [7]. In addition to its research implications, this finding may also affect clinical care. When studies are poorly conducted, they can lead to erroneous conclusions that may affect patient treatment. Such was the case in the early stages of the pandemic, when sporadic publications (later refuted) called for using azithromycin and hydroxychloroquine to treat critically ill patients [8–10]. This issue can be further exacerbated by the extraordinarily high level of interest in the pandemic and the ability to disseminate information around the world literally in a matter of seconds. It has been shown in other fields of medicine that during the time of urgency that was presented by the COVID-19 pandemic, there has been a shift away from the EBM standards [3, 4, 6]. There have been voices calling to reconsider the traditional model of EBM in the wake of the pandemic, and that the need for physicians to be agile and stay updated may sometimes stand in contradiction with the traditional EBM approach [11]. Although there are clear benefits for the rapid dissemination of information, its downsides must also be noted and studied.

We found no publications addressing the change in the quality of EBM in the ophthalmology literature during the COVID-19 outbreak. In our work, we examined the temporal evolution of EBM quality and standards for publishing in the field of ophthalmology during the COVID-19 pandemic in early 2020.

## 2. Methods

The COVID-19 pandemic erupted in late 2019, and by the end of September 2020, the vast majority of places globally have removed lockdown measures and adapted a pandemic routine [12]. We thus decided to acknowledge the end of September 2020 as marking the end of the outbreak phase of the pandemic. On September 30, 2020, we performed a comprehensive literature search of the PubMed and Scopus databases using the following search string: ((ophthalmology) OR (eye) OR (ocular)) AND ((corona) OR (COVID-19) OR (pandemic)). The initial results were then reviewed, and publications that did not pertain to both COVID-19 and the field of ophthalmology as well as non-English publications were excluded.

The remaining publications were included in our analysis and were graded using a commonly accepted categorization system developed by the Centre for Evidence-Based Medicine (CEBM) [13]. In brief, the highest level (level 1) was assigned to randomized control trials (RCTs) and systematic reviews of RCTs; level 2 was assigned to cohort studies and systematic reviews of cohort studies; level 3 was assigned to case-control studies and systematic reviews of these studies; level 4 was assigned to case series; and the lowest level (level 5) was assigned to case reports, expert opinions, editorials, and letters to the editor.

Each publication was graded as level 1 through 5 by two independent experienced ophthalmologists (authors EW and JL, with an experience as ophthalmologists of 10 and 24 years, respectively). The coefficient of agreement between the two graders was calculated, and disagreement between graders was discussed until consensus was reached.

For each publication, we documented the principal country of origin, journal, month of publication, number of citations, interval in days between submission and acceptance for publication, and whether the authors were required to revise the original manuscript. If more than one date relating to publication was available, we used the date of publication provided on PubMed as the date of acceptance. For each journal, we also obtained the 2019 impact factor and quartile from Web of Science [14].

### 2.1. Statistics

Analyses were performed using SPSS, version 25.0 (IBM Corp., Armonk, NY). Frequency counts and percentages generated were appropriate. After testing for normality, groups were compared using a paired Student correlations and analyzed using Pearson's correlation coefficient. Statistical significance was defined as *p* < 0.05.

## 3. Results

**Figure 1:**
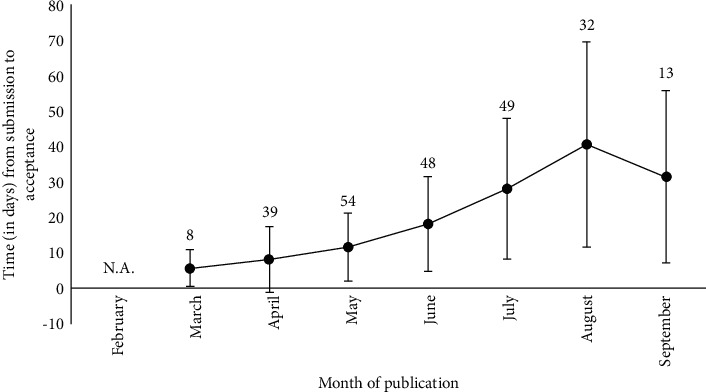
Time course of the mean ± SD interval (in days) between the initial submission and acceptance, by month of publication. Number of publications with available data added besides each time point.

Our search yielded 425 unique articles that met the inclusion criteria and were published between February 2020 and September 2020. These 425 articles originated from 35 countries, with the most articles originating from the US (15.5%), followed by India (13%), the UK (8.7%), China (6.8%), Italy (6.4%), and Hong Kong (4.5%). These 425 articles were published in 115 journals, with the most articles published in the Indian Journal of Ophthalmology, Eye, Ophthalmology, Graefe's Archives of Ophthalmology, and Acta Ophthalmologica. The mean (±SD) impact factor of these 115 journals was 2.24 ± 3.46 (range: 0.18–60.39). The interval between the first submission and acceptance for publication as well as information regarding whether revisions were required was available for 245 publications (57.6%). The mean interval between submission and acceptance was 20.4 ± 20.2 days, with 83 articles (19.5%) accepted within 7 days of submission. From February 2020 through September 2020, this interval increased significantly (Figure 1, Pearson's rho = 0.515, *p* < 0.0005). Of the publications for which the data were available, nearly half (48.2%) were accepted for publication without revision, and this did not change significantly from February through September (Table 1, Pearson's rho = 0.616, *p*=0.104).

**Figure 2:**
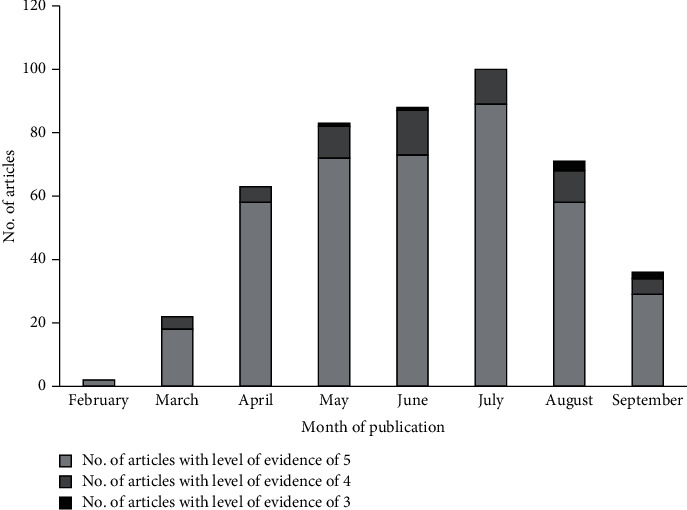
Summary of the level of EBM of the articles included in our analysis, by month of publication. Note that level 5 corresponds to the lowest level of EBM.

With respect to the level of EBM in the 425 publications, we found high inter-rater reliability between the two raters, with an interclass correlation coefficient of 0.718 (95% CI: 0.659–0.767, *p* < 0.0005). The majority of publications (359, consisting 84.5%) were rated the lowest at level 5, while 59 publication (13.9%) were rated as level 3, and 7 (1.6%) of publications were rated as level 3. None of the publications was rated at either level 2 or level 1, and no level 3 publications were accepted in February, March, or April 2020 (Figure 2).

We then examined the percentage of publications that were either level 4 or level 3 out of all publications accepted in a given month and found that this percentage increased significantly from February to September (Pearson's rho 0.108, *p*=0.024).

**Figure 3:**
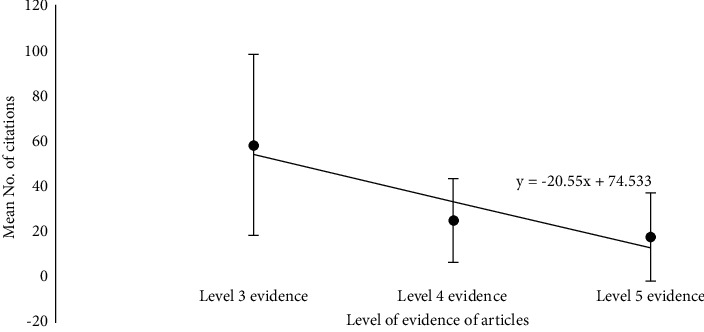
The mean ± SD number of citations per article plotted against the level of EBM. Articles with a higher level of EBM contained significantly more citations (rho = 2.44, *p* < 0.0005).

In addition, we found a significant positive correlation between the average number of citations per publication and the level of EBM (Figure 3); specifically, papers with higher levels of EBM included more citations (Pearson's rho 2.44, *p* < 0.0005). In contrast, we found no significant correlation between the average number of citations per publication and the month of publication (Table 2). Finally, we found no significant correlation between the average number of citations and either the impact factor or the ranking quartile of the journals in which the articles were published.

## 4. Discussion

Here, we report that in the early months of the COVID-19 pandemic, a relatively high proportion of COVID-19-related ophthalmology publications with a low level of EBM were accepted, and these articles were accepted in a relatively short period of time after submission. Moreover, we found that over the ensuing months an increasing percentage of articles with higher-level EBM were published, and the time between submission and acceptance increased.

These findings are not necessarily unexpected, given the understandable need in early 2020 to rapidly publish new information regarding this new pandemic that quickly disrupted our daily lives. Over time, the drive to publish papers with higher levels of EBM was met with a growing number of publications, which is reflected in the significant correlation between the number of citations per publication and the level of EBM.

Although the EBM levels of these publications increased significantly over time, it is important to note that no highest-level EBM papers (i.e., level 2 or level 1) in ophthalmology regarding COVID-19 were published in the first 8 months of the pandemic. This is not surprising, given that properly conducted prospective cohort studies and RCTs require both time to conduct and a foundation of knowledge on which to expand.

At the start of the pandemic, ophthalmologists needed to make countless clinical decisions, with implications for both their own well-being and their patients' outcome. However, these clinicians were largely baffled by the hastily published information—some of it conflicting—regarding questions such as, is it possible to contract the virus by contact with the patient's tears? How safe is it safe to perform nonurgent ocular surgery? Do we need to wear a facemask in public places? Indeed, one of the most famous victims of the pandemic was Chinese physician Dr. Li Wenliang, an ophthalmologist, who was among the first to report the outbreak of this new disease entity in Wuhan, China, later contracting the disease and subsequently dying from it [15].

The trend we found to rush and publish data applying lower publication standards is in no way unique to Ophthalmology and has also been noted in publications in other fields of medicine as well. Palayew et al. have found a significantly shorter latency between submission and acceptance of publications on the topic of COVID-19 when compared to prepandemic publications on other topics [16]. This emphasizes that the need to acquire knowledge on this new entity was universal and not unique to the field of ophthalmology.

As mentioned above, the approach to treating patients with hydroxychloroquine highlights the need to properly weigh the available evidence. Hydroxychloroquine was first regarded as a potentially lifesaving medicine for treating and/or preventing COVID-19; however, its use outside of controlled clinical trials was later discouraged with leading medical journals having to retract previously published articles [9, 17]. Moreover, in the early months of the pandemic, the lack of high-level EBM reduced the decision-making process to essentially the flip of a coin. However, as our analysis revealed, over time articles based on higher-level EBM are published, which in turn can enable clinicians to appropriately weigh conflicting data and make more informed decisions.

Unfortunately, our modern world and global lifestyle increases the likelihood of future pandemics. Therefore, the medical community should learn from our experiences with the COVID-19 pandemic, for example, by avoiding rushing to publish relatively low-quality articles, despite the temptation to fill the urgent need for information. Even in the face of a pandemic, standards must be maintained, and adhering to these standards will ultimately save more lives compared to the rapid dissemination of low-quality information. Unfortunately, however, maintaining these standards is increasingly difficult in our modern fast-paced Internet age.

The main strengths of our study are that it analyzes the quality of publications in the field of ophthalmology during the early stage of the COVID-19 pandemic. No results were excluded, and we used two independent graders to assess the EBM level of each publication.

Our study has several limitations that warrant discussion. First, our analysis was limited to publications written in English; therefore, including non-English publications may have provided a more accurate picture of the articles available to clinicians in non-English-speaking countries. Second, other methods are available for categorizing EBM [18], and the possibility of a bias by graders when ranking the publications cannot be fully excluded. However, the method that we used is generally well-accepted and consists of accepted parameters for ranking [19]. In addition, the method we used is easy to implement and is reproducible, given the high inter-rater correlation.

In conclusion, our findings indicate that in the field of ophthalmology, the desire to quickly disseminate knowledge regarding COVID-19 inevitably resulted in the publication of a high percentage of papers with lower levels of EBM, with relatively lower publication standards; however, as time passed, these standards increased, resulting in the publication of articles with higher levels of EBM.

## Figures and Tables

**Table 1:** Summary of the articles published without revision and articles published with at least one revision, by month of publication.

	Number of articles published per month	Published after revision (%)	Published without any revision (%)	Data unavailable (%)
February	2	0	0	2 (100)
March	18	5 (27.8)	3 (16.7)	10 (55.5)
April	58	22 (37.9)	17 (29.4)	19 (32.7)
May	83	25 (30.1)	29 (34.9)	29 (34.9)
June	88	21 (23.9)	27 (30.6)	40 (45.5)
July	89	27 (30.3)	22 (24.8)	40 (44.9)
August	58	16 (27.6)	16 (27.6)	26 (44.8)
September	29	10 (34.5)	3 (10.3)	16 (55.2)
Total	425	126 (29.7)	117 (27.5)	182 (42.8)

**Table 2:** The mean ± SD number of citations per article, by month of publication.

	Mean number of citations
February	15 ± 14.14
March	21.67 ± 27.46
April	17.51 ± 20.6
May	18.02 ± 19.6
June	19.22 ± 20.64
July	16.87 ± 15.29
August	19.9 ± 24.81
September	26.83 ± 22.67

Data are presented as the mean ± SD.

## Data Availability

The data used to support the findings of this study are available from the corresponding author upon request.
